# Does a reclined backrest with less legroom meet the same comfort as a fixed backrest with 80 mm more leg room?

**DOI:** 10.3233/WOR-230643

**Published:** 2024-06-25

**Authors:** Sander Maxim Eversdijk, Frederik Johannes Cornelis de Vos, Aldo Aaldert Theoduros van Zee, Nola Cornelia Adriana Houtepen, Mily Isabelle van Haaff, Maxime Albertine Corelijne Iserief, Peter Vink

**Affiliations:** ^1^ TU Delft Faculty of Industrial Design Engineering, Delft, The Netherlands

**Keywords:** Posture, ergonomics, sitting position, travel, surveys & questionnaires

## Abstract

**Background:**

In vehicles there is often limited space for seats. This might mean that reclining the back rest reduces the legroom. The second row in a cargo van has this problem and in this limited space an upright seat and a reclined seat with less legroom was developed and tested.

**Objective:**

The research question of this study is: *Does a reclined backrest with less leg room result in the same comfort and/or discomfort as an upright backrest with more leg room?*

**Methods:**

Twenty participants are asked to sit 45 minutes in the upright seat with 8 cm more legroom and 45 minutes in the reclined seat. Ten participants started in the upright seat and ten in the reclined. Participants had to complete a comfort and discomfort questionnaire every 15 minutes and a qualitative interview was conducted after experiencing both seats.

**Results:**

For comfort no statistically significant differences were found between both seats. For discomfort statistically significant differences were found where discomfort was lower in the reclined seat. Half of the participants preferred the upright and half the reclined seat. The interviews showed that the reclined position was more related to relaxation.

**Conclusions:**

This study indicates that a more reclined back rest results in less discomfort, but that does not lead to a clear preference of participants. The reclined position is associated with relaxing, and this study indicates that for the relaxing state the more reclined seat is preferred. For more active situations the upright posture seems better.

## 1. Introduction

Cars are not only an important means of transportation, but they also play a crucial role in the economy, the environment and society as a whole.^1^ Passengers travelling in cars, trains, or planes are often confronted with lack of legroom and the seat adjustment settings. Some studies clearly show that less legroom leads to less comfort. Anjani et al.^2^ showed for instance that the comfort at a pitch of 28” (seat pitch is measured from a point on a seat to the exact same point of the seat in-front/behind it) resulted in a comfort score around 4 on a scale 0–10, while a pitch of 34” resulted in a comfort score of around 8 on the same scale. Vanacore et al.^3^ found different comfort values for different recline positions. Vink^4^ describes differences in comfort with different back rest angles and for driving or sitting in a rear seat. Occupants chose the 117 degrees reclined back rest. As many vehicles have limited space reclining might mean that the leg space is reduced. Therefore, in this research we study if it is more appreciated by occupants to have more leg room or more recline.

In this research, a second row of seats for commercial vehicles is developed that has the same appearance as the front row seats, but adds extra flexibility, comfort and functionalities. Legislation for cargo vans reduces the design space as the cargo space should have a certain size. For instance, private motor vehicle and motorcycle tax^5^ requirements for delivery vans state that the cargo area is at least 130 cm high over a width of at least 20 cm and over a length of at least 150 cm. Additionally, the front row cannot be changed as these are determined by the original equipment manufacturer (OEM) like Mercedes or Volkswagen. However, within the limited space it is possible to add a reclining mechanism. The seat cannot translate backwards to keep the cargo space, meaning that the reclined version of the seat has to move forward and will reduce legroom. This principle has been applied in aircraft seats (e.g.,^6^) where it was found that limited legroom does not significantly contribute to the perception of comfort or discomfort and does not result in significantly reduced neck, lower back, hamstring range of motion or flexibility. According to Porta^7^ the minimum seat space should be between 68.1 and 70.1 cm. The relevancy for this research is that we preferably should not go past this value. Reclining could increase the comfort^,8^ but in our case it does reduce the leg room. There is literature which states that there is a difference in comfort and discomfort experience^.9^ Comfort is seen as pleasant state or relaxed feeling of a human being in reaction to its environment and Discomfort is seen as an unpleasant state of the human body in reaction to its physical environment.^10^ Therefore, in this study not only comfort is measured, but discomfort as well. To our knowledge, there is no literature studying the effect on (dis)comfort of improving the recline of the backrest in combination with reducing leg room.

The research question studied in this paper is:

*Does a reclined backrest with less leg room result in the same comfort and/or discomfort as an upright backrest with more leg room?*

Our hypothesis is that a reclined backrest with less legroom will show a different comfort and discomfort score than a fixed backrest.

## 2 Methods

### 2.1 Participants

To study the effect of change in recline and leg room twenty participants are asked to sit 45 minutes in an upright seat with 8 cm more legroom and 45 minutes in the reclined seat. In science usually a population of P5 to p95 is chosen.^11^ However, the users of the product (cargo van) are usually craftsmen. An assumption was made that craftsman generally are larger in size due to the physical nature of the job. Another assumption made is that women can also be handymen. Additionally, it is assumed that shorter participants would not experience the reduced leg room so much. Therefore, the experiment will be done with both P50-P95 males and females, with genders split among participants equally. Relevant anthropometric data were recorded of all 20 participants (buttock knee depth, popliteal length, hip width, weight, stature, shoe size, see Table 1). The sampling method will be convenience sampling. The sample is representative for our sample group because the vans are not only used to drive employees to a worksite, but also for family purposes, like vacations or picking up children at school. The occupation of our user group was not a selection criterium as we assume it is not so relevant. The recruitment was done merely on the criteria of height.

**Table 1.** Participants.

Participant (m/f)	Buttock knee depth mm	Popliteal Length mm	Hip width mm	Weight kg	Total length mm	Shoe size (EU unit)
1 f	501	510	310	55.3	1691	39.5
2 m	532	540	500	110.1	1924	46
3 m	570	570	450	89.7	1918	45.5
4 f	554	520	445	76.3	1816	43.5
5 m	520	515	365	75.5	1855	44
6 m	570	550	395	86.3	1929	44
7 f	525	484	400	62.2	1731	36
8 f	494	499	410	72.7	1742	40.5
9 m	525	493	381	81.5	1917	46
10 f	520	431	394	78.3	1697	39
11 m	565	455	374	70.5	1793	42
12 m	531	512	412	90.5	1847	41.5
13 f	473	471	368	59.1	1670	39
14 f	534	471	363	77.2	1810	41
15 m	516	501	364	73.8	1900	44
16 m	530	493	433	110.6	1754	42
17 f	491	476	383	59.5	1766	42
18 m	523	523	358	93.3	1849	44.5
19 f	503	456	360	58.3	1680	38
20 f	538	501	428	91.5	1725	41
Average	525.8	498.6	394.7	78.6	1800.7	42
Standard deviation	26.0	33.8	42.2	16.0	88.4	2.77

### 2.2 Protocol

Four second row car seats were manufactured (see Fig. 2). The test is conducted using four car seats, two with the reclined back rest and limited leg room and two with the upright back rest and more leg room. The participants are divided into two groups, one group starting upright and one group starting reclined. This way, 4 participants could participate in a 110-minute session (including preparation and break). Prior to the test, the participants were asked to complete an informed consent form.

The first two regular seats have a seating angle of 116 degrees and a legroom of 750 mm, this length includes the seat depth of 500 mm. These are called regular as these seats are now available in cargo vans for the second row. The second two adjusted seats have a seating angle of 133 degrees and a legroom of 670 mm with the same seat depth. Previous research has shown that more than 5 degrees adjustment can lead to different comfort values.^11^ Therefore, it is assumed that an adjustment of 17 degrees might be sufficient to experience a difference. The legroom space is based on what is possible in the space of the cargo van and it is comparable to the study of,^2^ who had a leg room (seat depth + space between bull nose and the seat in front) of 640 mm for the 28” pitch and 690 mm for the 30” pitch seat. The participants were asked to sit for 45 minutes on their assigned first seat, walk 10 minutes and then sit 45 minutes on the other seat. Prior to the test the participants were not told what the difference in the seat is, they just were asked to have a seat.

**Figure 1.**
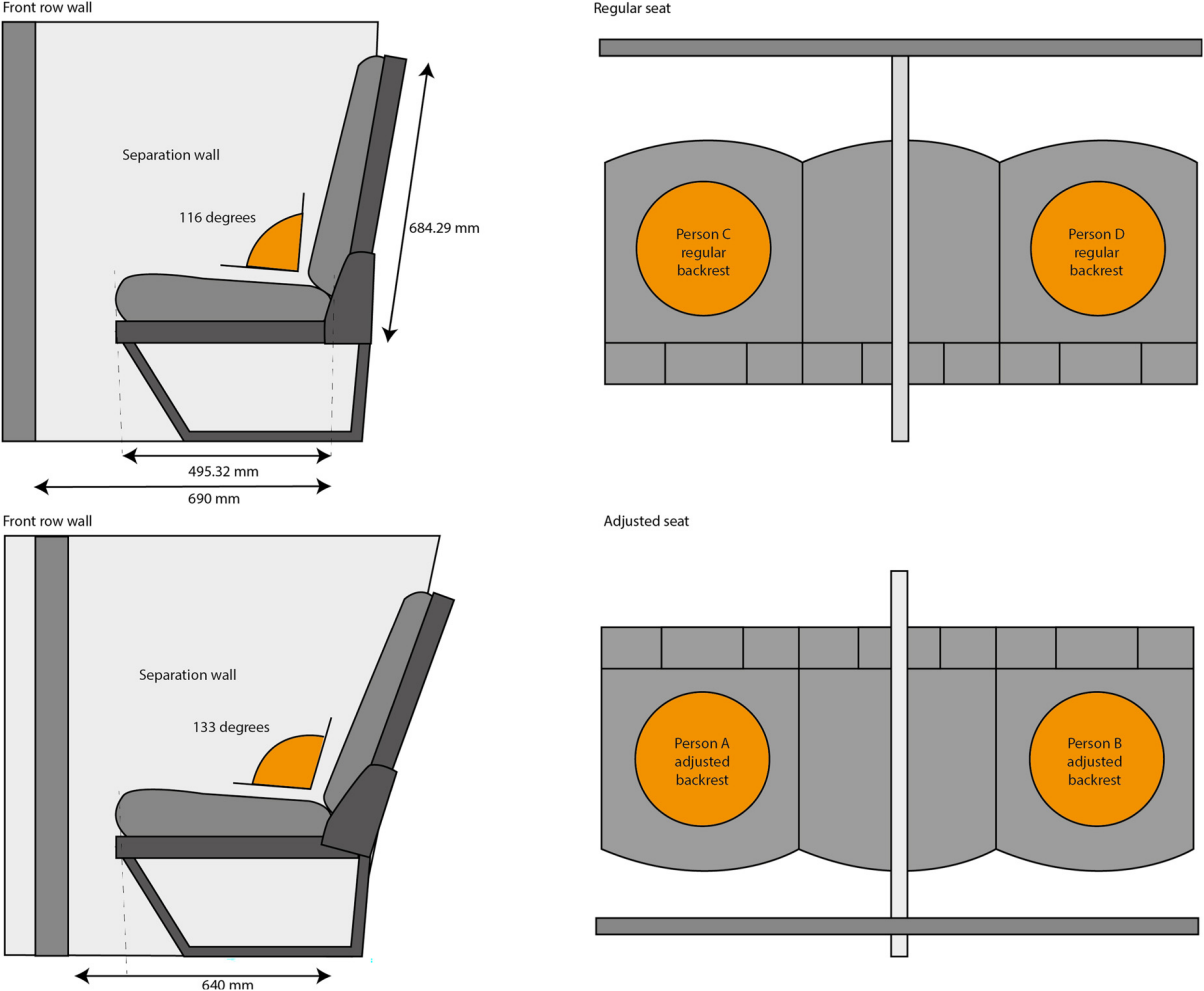
Test setup.

**Figure 2.**
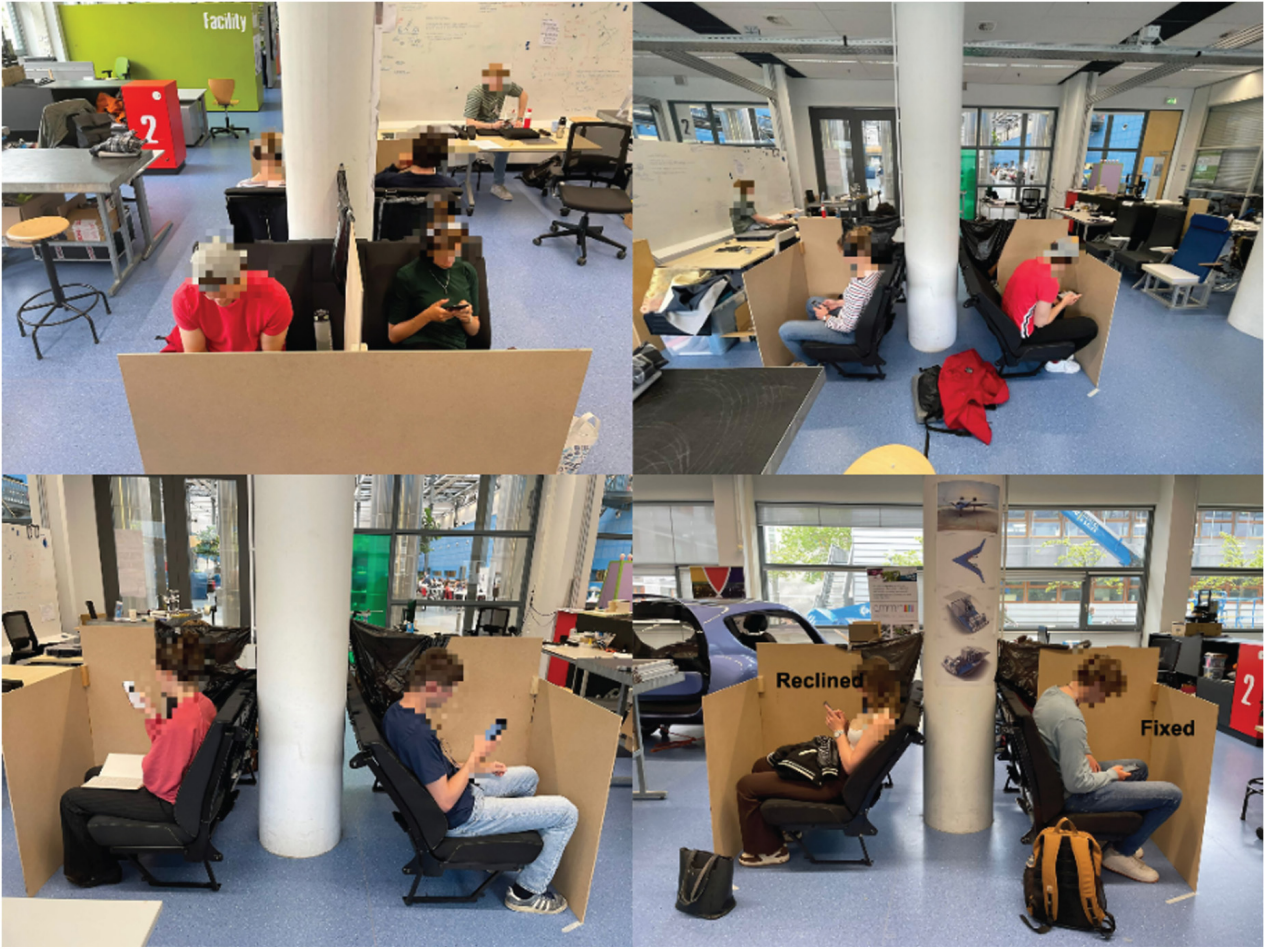
Test setup with participants.

Piro et al.^12^ found that the quality of conversation is strongly correlated to rating comfort. Also, Hiemstra-van Mastrigt^13^ reported the influence of a conversation on comfort. To prevent this effect participants were instructed not to talk, and a separation wall was placed over the middle seat of both seating setups to avoid other contact. Figure 1 shows the test setup. The setup was positioned at the Applied Labs of the faculty of Industrial Design Engineering of the Delft University of Technology. Figure 2 shows the setup with the participants.

The participants were asked to complete a questionnaire after 15 minutes, 30 minutes and at the end of the experiment (45 minutes). It consisted of questions on comfort, discomfort, how they feel, and how they have experienced the seat (See Appendix A). The user was allowed to do what he/she wanted like reading or watching a video, but they should do the same activity in both seats. As a compensation participants received a voucher of 20 euros for participating. First, a pilot test was conducted using people that walked along the set up. In the pilot we measured if people felt which seat, was the reclined seat. Five people sat down and were asked about the experienced seating angle. A difference was felt, which confirmed the functionality of the set up and supported the decision to continue with the research. The reduction in leg room was also noticed.

### 2.3 Data collection

Participants had to rate the comfort and discomfort on a Likert scale 0 to 10. A paired comparison was made using Wilcoxon test (*p* < .05) to process the data.

Two rank totals— one for each seat— are generated once the data is ranked. The smaller of the rank totals is the Wilcoxon test statistic, or “W”. The less likely it is to have happened by coincidence, the smaller it is (considering the number of participants).^14^ How likely it is to obtain your particular value of W purely by chance, is shown in a table of critical values of W. To analyse the data, three Wilcoxon tests are conducted, one at each fifteen minutes that data is recorded. This is done for comfort as well as for discomfort, resulting in six Wilcoxon tests in total.

Data and results from the questionnaire were collected and analysed using IBM^®^ SPSS^®^ Statistics version 26.

Apart from the total comfort and discomfort questions, the participants were asked to complete the local postural discomfort questionnaire according to the approach of Grinten and Smitt^15^ each 15 minutes.

After experiencing both seats an interview with the participants was organized gaining information on their preferences and reasons for the preference.

## 3 Results

### 3.1 Comfort and discomfort analysis

**Figure 3.**
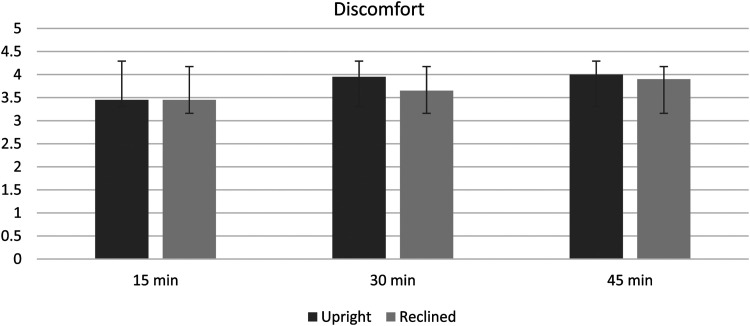
Discomfort values on three recorded moments in time.

For Comfort no statistically significant differences were found between setup A and B. The comfort scores in the upright position were 6.7, 6.3 and 6.25 at 15, 30 and 45 minutes respectively and for the reclined 6.85, 6.45 and 6.25 at 15, 30 and 45 minutes respectively. For Discomfort statistically significant correlations were found between upright and reclined setup A and B. In the reclined position the discomfort is significantly lower at all times. There is a slight increase in discomfort over time in both the upright and reclined position (see Fig. 3). This phenomenon is described in the literature as well(e.g.,.^16^)

In Fig. 3 the discomfort values averaged over the 20 participants for the three recorded moments in time (15, 30 and 45 minutes) for the upright (light grey) and reclined (dark grey) situation on a scale 0– 10 (0 = no discomfort at all; 10 = extreme discomfort).

**Figure 4.**
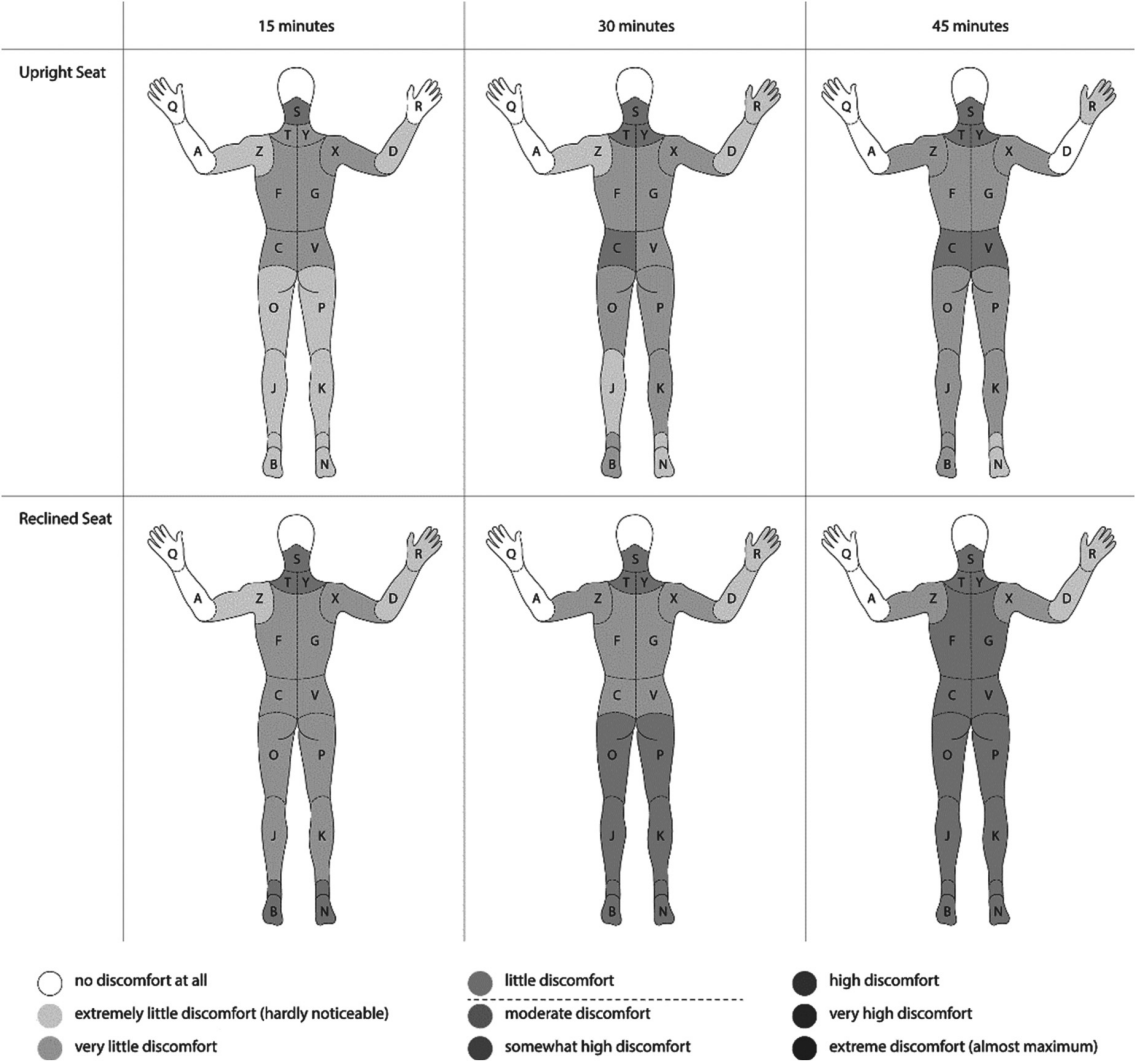
Local Postural Discomfort (LPD) scores averaged over all participants for 15, 30 and 45 minutes in both seats.

Region-specific Local Postural Discomfort (LPD) values are shown in Fig. 4. Data used in this Figure differ and was obtained with LPD-questions rather than a straightforward discomfort score question. As represented by the Figure, results show that in general, there are more regions with discomfort in the reclined seat.

**Table 2.** Comfort analysis values.

Timepoint	15 minutes	30 minutes	45 minutes
Z	–,414	–,447	,000
*p*-value	,679	,655	1,000
W Stat vs. W Crit	68 > 34	33,5 > 13	39 > 13

**Table 3.** Discomfort analysis values.

Timepoint	15 minutes	30 minutes	45 minutes
Z	–2,941	–2,777	–3,286
*p*-value	,003	,005	,001
W Stat vs. W Crit	18 < 40	26 < 46	17 < 52

When taking a closer look, Fig. 4 also shows that for both seating setups, the neck (area’s S, T, and Y) is one of the area’s where participants felt the most discomfort. Within the first 30 minutes, the two seating angles score relatively similar in the upper body. One thing that does stand out in the upper bodily discomfort is the lower back (left side C), which has a higher rating of discomfort (little discomfort) in the upright seat after 30 minutes. A bigger difference becomes visible for the lower body: higher levels of discomfort are experienced (also little discomfort) here, by participants sitting in the reclined seat from 15 minutes onwards. After only 15 minutes in the reclined seat there is higher discomfort in the feet than in the upright seat and this discomfort persists for the time remaining. After 45 minutes, the reclined seat is associated with more discomfort in both upper and lower body. However, this is in contradiction with finding that the total discomfort is higher in the upright position (see Figure 4). Probably the early (little discomfort after 30 minutes) lower back region discomfort (area C) in the upright seat is so annoying that the total discomfort score is higher for the upright situation.

The results of the qualitative interview after sitting on both seats can be found in Tables 4 and 5.

**Table 4.** Comments mentioned in the qualitative interview that was conducted after experiencing both seats. The explanation for the preference is given under the first two rows.

Upright	Reclined
*6 out of 10 participants that started in the fixed setup preferred the reclined seat*	*4 out of 10 participants that started in the reclined setup preferred the reclined seat*
A1: Reclined. *The reclined seat ended without backpain, also the neck and feet complaints were less.*	B1: Reclined. *More room to move and lower back support in my opinion.*
A2: Reclined. *The reclined seat has a more relaxed position for the back, not for the shoulders, but it still is the better one.*	B2: Upright. *Hard to enjoy the reclined seat due to cramped legs. Upright seat has more legroom which was nice, and I could decide myself how to sit (leaning backwards or sitting upright).*
A3: Reclined. *The upright seat is better for the back. I prefer the relaxed seat for longer periods of time, for shorter periods the upright seat is better.*	B3 Upright. *First the relaxed seat was better, but it brought discomfort in my back leading to more discomfort in the long run. Upright wrong at first but saves the back from fatigue.*
A4: Upright. *Because it was the first one, I sat on.*	B4: Reclined. *Less pain in neck and back and feels more comfortable overall.*
A5: Reclined. *The upright seat causes more stress in my lower back. This disappears with the reclined seat. The upright seat also causes more neck pain.*	B5: Upright. *Looking at the seat itself, the reclined is better, however there was too little legroom so that I couldn*’*t even place my feet straight and I got cramps in my legs and hips. So upright is better due to more legroom. Neck hurt more with reclined seat due to looking down on my phone.*
A6: Upright. *Because it is much more relaxed than the reclined seat.*	B6: Reclined. *My neck got heavy in the reclined seat. The reclined seat was lovely. Tailored exactly to my body length.*
A7: Reclined. *Because I can sleep easier in the reclined seat*	B7: Reclined. *Because I could relax more.*
A8: Upright. *Because the reclined seat has less space for legs/feet. I am missing the headrest with the reclined seat as well. Maybe if it were there, I would have preferred the reclined seat.*	B8: Upright. *I only suffered a bit from neck pain because there was no headrest, also due to looking on my phone. If I looked in front of me, it was better for my neck.*
A9: Upright. *The reclined seat had too little space for feet.*	B9: Upright. *Better to sit upright due to the fact that spine was more aligned all the way up.*
A10: Reclined. *Because I experienced less discomfort in the back.*	B10: Upright. *With the reclined seat I suffered more from neck pains, this costed more energy. Upright seat has more space for feet.*

**Table 5.** Extra comments within the qualitative interview.

Fixed	Reclined
A1: Reclined. *I am missing the headrest for both the seats; it causes neck pain.*	B1: Reclined. –
A2: Reclined. *A headrest would be premium for a powernap in the reclined seat.*	B2: Upright. *It is possible to rest a foot on one knee in the upright seat, this can take away pressure from the lower leg. My calves were tight because of the reclined position because I had to place my feet back underneath the seat.*
A3: Reclined. –	B3 Upright. –
A4: Upright. *The upright seat had more legroom, which I needed because my legs started cramping.*	B4: Reclined. *I suffer from some muscle pain in my legs and because of this the seats feel harder than normal, and which is also why I had more complaints with my legs, so I rated them higher.*
A5: Reclined. *I would have liked extra legroom in the lying position.*	B5: Upright. –
A6: Upright. *The back resistance is a bit too high: for me it would have been better if it was 3*– *4* *cm lower.*	B6: Reclined. –
A7: Reclined. *Well organised and thanks for the voucher*	B7: Reclined. *Maybe measure the fatigue in the knees too.*
A8: Upright. *With placement of car door and back window it would have been easier to give feedback. Both have good support for the back which I enjoyed.*	B8: Upright. *Headrest would be nice.*
A9: Upright. –	B9: Upright. –
A10: Reclined. *Some neck support would be nice in the reclined backrest.*	B10: Upright. *Seems like with both seats the right shoulder is less comfortable and blocked. Maybe it would be practical if I would have sat on the outer seat as well.*

During the test, pictures of the participants were taken. The pictures can be found in Fig. 2, p. 5. Most of the participants used their smartphone during the test. All participants exhibited behaviour which can be performed in a car as well.

The qualitative data (see Table 4) show that 10 participants prefer the upright and 10 the reclined seat. The arguments differ. The reclined seat was often experienced as more relaxed and for the upright seat the legroom was appreciated. In both seats participants preferred to have a headrest, which is in line with the higher LPD values in the neck region.

## 4 Discussion

The main research question was if a reclined backrest with less leg room results in the same comfort and/or discomfort as an upright backrest with more leg room. The comfort did not differ significantly, but the discomfort did. According to Zhang et al.^17^ discomfort is more related to physical factors and comfort more to emotional factors. In this case the physical environment is adapted, and it seems reasonable that discomfort is especially influenced. The discomfort is less in the reclined position, which would suggest that within the same space it makes more sense to add recline. However, this difference has no influence on the preference, which makes a hard statement questionable. The ‘upright’ seat in this experiment is rather reclined as other studies^18, 19^ show that a 117–119 degrees back rest recline is preferred by drivers and passengers in the rear seat. On the other hand, for relaxing and sleeping back rest angles of more than 120 degrees are preferred (e.g.,^8, 16^). This was also the comment in the open interviews that reclined is more associated with relaxing.

This shows that the comfort and discomfort experience is dependent on the task. Smulders et al.^16^ also showed in a business class seat that for active sitting 113 degrees is preferred, for passive sitting 121 and for sleeping 146 degrees. This might hint towards dynamic seats, where users can adapt the back rest angle. Users can determine their own seating angle and legroom, and more easily change their seating position (micro movements) which also provides more comfort.^20^

There might be a difference between participants: for instance, those who consider a backrest recline more important than foot and legroom space. This could explain the fact that an even number of participants preferred one of the seats.

It is also clear from the interviews that the headrest is a part among all participants that needs attention, which is also reflected in the LPD map. There was no headrest was present in both set ups.

Another interesting finding is the moderate discomfort in the right hand indicated by all participants. This could be explained by the phone use that most participants did with their right hand, which is in accordance with a study of Udomboonyanupap et al..^21^

The participants in the reclined seat all experienced discomfort in their feet due to the 8 cm less leg room. The other local postural discomfort (LPD) results are a bit difficult to understand as the total discomfort scores were lower in the reclined position, while the LPD data do not show this so clearly. The LPD was filled in on different screen size smartphones instead of a standardized format, which could have influenced the scoring of discomfort. A disadvantage of this project is that we used a wooden plate to limit the legroom. In a real situation, the front seats might have space under the seat where feet can be placed. In our setup the separation wall did not have this space.

Other limitation of the experiment could be that the test was done in a static environment, not a moving or simulated moving environment, that the number of participants was limited to 20 and the test setup was in a laboratory. The separation wall was not realistic in comparison to a normal seat in the front. On the other hand, it was a within subject design with the environment exactly the same for both conditions showing the effects between the two conditions having not much other interferences.

## 5 Conclusion

This study indicates that a more reclined back rest results in less discomfort, but that does not lead to a clear preference of participants. Many participants used a smart phone, which asks for a more upright posture, The reclined position is associated with relaxing, and this study indicates that for the relaxing state the more reclined seat is preferred. For more active situations the upright posture seems better. Ideally, participants can choose the back rest angle and adapt it to the activity they perform or their preferences.

## Supplemental Material

sj-docx-1-wor-10.3233_WOR-230643 - Supplemental material for Does a reclined backrest with less legroom meet the same comfort as a fixed backrest with 80 mm more leg room? Supplemental material, sj-docx-1-wor-10.3233_WOR-230643 for Does a reclined backrest with less legroom meet the same comfort as a fixed backrest with 80 mm more leg room? by Sander Maxim Eversdijk, Frederik Johannes Cornelis de Vos, Aldo Aaldert Theoduros van Zee, Nola Cornelia Adriana Houtepen, Mily Isabelle van Haaff, Maxime Albertine Corelijne Iserief and Peter Vink in WORK
