# Supplementary material for: Does a reclined backrest with less legroom meet the same comfort as a fixed backrest with 80 mm more leg room?
Source: Work. 2024 Jun 25;83(1):84–92. doi: 10.3233/WOR-230643 (PMC13365624; doi:10.3233/WOR-230643)
Supplement: sj-docx-1-wor-10.3233_WOR-230643 - Supplemental material for Does a reclined backrest with less legroom meet the same comfort as a fixed backrest with 80 mm more leg room? [file sj-docx-1-wor-10.3233_WOR-230643.docx]

# Appendix A: Questionnaire

1. Group A and B (either starts with leg room or starts with an angle)

Per time step

1. What is the comfort rating of your current sitting position?
   No comfort 0 -> 10 extreme comfort
2. What is the discomfort rating of your current sitting position?
   No discomfort 0 -> 10 extreme discomfort
3. LPD based on the image below; all areas (a-z) were ranked separately.


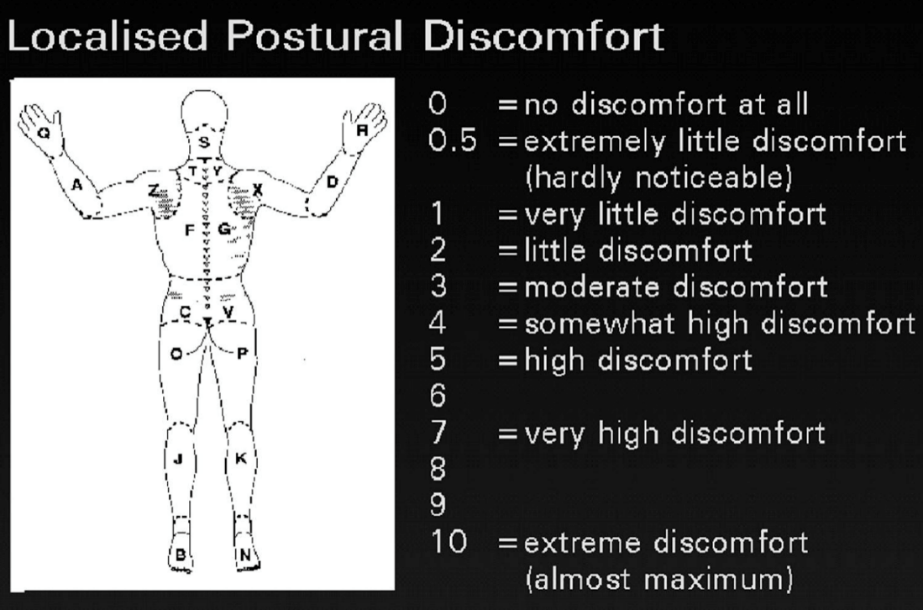


After all time steps

1. Which sitting position did you prefer?
   1. Upright
   2. Reclined
2. Please specify your answer
3. Open remarks

# Appendix B: Informed Consent

The relation between a reclined backrest and legroom.

This research is conducted as part of the MSc IPD study Industrial Design Engineering at TU Delft.

**Students**: Maxime Iserief, Mily van Haaff, Aldo van Zee, Nola Houtepen, Rik de Vos, Sander Eversdijk

**Contact person:** Rik de Vos, [f.j.c.devos@student.tudelft.nl](mailto:f.j.c.devos@student.tudelft.nl),

Informed consent participant

I participate in this research voluntarily.

I acknowledge that I received sufficient information and explanation about the research and that all my questions have been answered satisfactorily. I was given sufficient time to consent my participation. I can ask questions for further clarification at any moment during the research.

I am aware that this research consists of the following activities:

1. Interview
2. User test

I am aware that data will be collected during the research, such as notes, photos, video and/or audio recordings. I give permission for collecting this data and for making photos, audio and/or video recordings during the research. Data will be processed and analysed anonymously (without your name or other identifiable information). The data will only be accessible to the research team and their TU Delft supervisors.

The photos, video and/or audio recordings will be used to support analysis of the collected data. The video recordings and photos can also be used to illustrate research findings in publications and presentations about the project.

I give permission for using photos and/or video recordings of my participation:  
(select what applies for you)

- in which I am recognisable in publications and presentations about the project.
- in which I am not recognisable in publications and presentations about the project.
- for data analysis only and not for publications and presentations about the project.

I give permission to store the data for a maximum of 5 years after completion of this research and using it for educational and research purposes.

I acknowledge that a financial compensation in the form of a 20 euro VVV gift card will be provided for my participation in this research.

With my signature I acknowledge that I have read the provided information about the research and understand the nature of my participation. I understand that I am free to withdraw and stop participation in the research at any given time. I understand that I am not obliged to answer questions which I prefer not to answer and I can indicate this to the research team.

I will receive a copy of this consent form.

_________________________ _________________________

Last name            First name

___ / ___ / 2023 _________________________

Date (dd/mm/yyyy) Signature
